# Awards Recommended by the Committee of Distribution for the Year 1917

**Published:** 1917-08-11

**Authors:** 


### AWARDS RECOMMENDED BY THE COMMITTEE OF DISTRIBUTION FOR THE YEAR 1917.

#### THIRTY-FIVE GENERAL HOSPITALS.

Charing Cross Hospital, £1,465 16s. 3d.; French Hospital, £174 5s. 10d.; Great Northern Central Hospital, £1,567 12s. 11d.; Guy's Hospital, £2,107 3s. 4d.; Hampstead and N.W. London Hospital, £882 4s. 7d.; Italian Hospital, £143 18s. 9d.; Kensington General Hospital, £88 2s. 6d.; King's College Hospital, £2,093 9s. 2d.; King Edward's, Ealing, £161 11s. 3d.; London Hospital, £8,070 5s. 10d.; London Homœopathic Hospital, £753 19s. 2d. Phillips' Memorial Homœopathic Hospital, £52 17s. 6d.; London Temperance Hospital, £648 4s. 2d.; Metropolitan Hospital, £1,198 10s.; Mildmay Hospital, £280 0s. 10d.; Mildmay Memorial Hospital, £76 7s. 6d.; Miller Hospital and Royal Kent Dispensary, £615 17s. 11d.; Poplar Hospital, £742 4s. 2d.; Prince of Wales' Hospital, £1,079 0s. 10d.; Royal Free Hospital, £1,527 10s.; St. Andrew's, Dollis Hill, £27 8s. 4d.; St. George's Hospital, £2,615 7s. 1d.; SS. John and Elizabeth Hospital, £170 7s. 6d.; St. John's Hospital, Lewisham, £240 17s. 6d.; St. Mary's Hospital, £2,230 10s. 10d.; St. Thomas's Hospital, £812 14s. 2d.; Seamen's Hospital Society, £1,185 15s. 5d.; The Middlesex Hospital and Convalescent Home, £3,210 13s. 9d.; University College Hospital, £1,739; Walthamstow, etc., Hospital, £160 11s. 8d.; Wandsworth, Bolingbroke Hospital, £422 0s. 5d.; Wimbledon, £76 7s. 6d.; West Ham Hospital, £1,120 3s. 4d.; West London Hospital, £1,429 11s. 8d.; Westminster Hospital, £1,470 14s. 2d.

#### SIX CHEST HOSPITALS.

City of London Hospital for Diseases of the Chest, Victoria Park, £986 0s. 5d.; Hospital for Consumption, Brompton, £2,347 1s. 3d.; Mount Vernon Consumption Hospital, £123 7s. 6d.; Royal Hospital for Diseases of the Chest, £498 7s. 11d.; Royal National Sanatorium, £14 13s. 9d.; Royal National Hospital, Ventnor, £176 5s.

#### TWENTY CHILDREN'S HOSPITALS.

Alexandra Hospital for Hip Disease, £146 17s. 6d.; Banstead Surgical Home, £33 5s. 10d.; Barnet Home Hospital, £32 6s. 3d.; Belgrave Hospital, £332 18s. 4d.; Cheyne Hospital for Incurable Children, £195 16s. 8d.; East London Hospital for Children, £850 17s. 11d.; Evelina Hospital for Sick Children, £272 4s. 2d.; Home for Incurable Children, £39 3s. 4d.; Home for Sick Children, Sydenham, £147 17s. 1d.; Hospital for Sick Children, £1,399 4s. 7d.; Infants' Hospital, Westminster, £285 18s. 4d.; Kensington, for Children, and Dispensary, £74 8s. 4d.; Paddington Green Hospital for Children, £357 7s. 11d.; Queen's Hospital for Children, £1,460 18s. 4d.; Royal Waterloo Hospital for Children and Women, £599 5s.; St. Mary's Hospital, Plaistow, £301 11s. 8d.; St. Monica's Hospital, Brondesbury, £86 3s. 4d.; Victoria Hospital for Children, Chelsea, £687 7s. 6d.; Victoria Home, Margate, £44 1s. 3d.; Hospital for Hip Disease, Sevenoaks, £48 19s. 2d.

#### EIGHT LYING-IN HOSPITALS.

British Home for Mothers and Babies, £37 4s. 2d.; City of London Lying-in Hospital, £195 16s. 8d.; Clapham Maternity Hospital and Dispensary, £25 9s. 2d.; East End Mothers' Home, £110 12s. 11d.; General Lying-in Hospital, Lambeth, £203 13s. 4d.; Jewish Maternity, £48 19s. 2d.; Plaistow Maternity Hospital, £54 16s. 8d.; Queen Charlotte's Lying-in Hospital, £550 5s. 10d.

#### SIX HOSPITALS FOR WOMEN.

Chelsea Hospital for Women, £390 13s. 9d.; Hospital for Women, Soho Square, £432 15s. 10d.; Grosvenor Hospital for Women and Children, £155 13s. 9d.; New Hospital for Women, £554 4s. 2d.; Samaritan Free Hospital, £521 17s. 11d.; South London Hospital, £109 13s. 4d.

#### TWENTY-THREE OTHER SPECIAL HOSPITALS.

Middlesex Hospital, Cancer Wing, £277 2s. 1d.; London Fever Hospital, Islington, £244 15s. 10d.; Gordon Hospital for Fistula, £19 11s. 8d.; St. Mark's Hospital for Fistula, £386 15s. 5d.; National Hospital for the Diseases of the Heart, £127 5s. 10d.; Female Lock Hospital, Harrow Road, £358 7s. 6d.; Hospital for Epilepsy, Paralysis, etc., £262 8s. 4d.; National Hospital for the Paralysed and Epileptic, £1,101 11s. 3d.; West End Hospital for Diseases of the Nervous System, £340 15s.; Central London Ophthalmic Hospital, £195 16s. 8d.; Royal Eye Hospital, £255 11s. 3d.; Royal London Ophthalmic Hospital, £1,057 10s.; Royal Westminster Ophthalmic Hospital, £135 2s. 6d.; Western Ophthalmic Hospital, £115 10s. 10d.; Royal National Orthopædic Hospital, £550 5s. 10d.; Royal Sea Bathing Hospital, Margate, £97 18s. 4d.; St. John's Hospital for Diseases of the Skin, £39 3s. 4d.; St. Peter's Hospital for Stone, £140 0s. 5d.; Central London Throat and Ear Hospital, £30 7s. 1d.; Throat Hospital, Golden Square, £100 17s. 1d.; London Throat Hospital, £44 1s. 3d.; Metropolitan Ear, Nose, and Throat Hospital, £63 12s. 11d.; Royal Dental Hospital of London, £179 3s. 9d.

THIRTY CONVALESCENT HOSPITALS.

Metropolitan Convalescent Institution, Walton, £366 4s. 2d.; Metropolitan Convalescent Institution, Broadstairs, £279 1s. 3d.; Metropolitan Convalescent Institution, Bexhill, £539 10s. 5d.; All Saints' Convalescent Hospital, Eastbourne, £53 17s. 1d.; All Saints' Convalescent Home, St. Leonards, £27 8s. 4d.; Ascot Priory Convalescent Home, £46 0s. 5d.; Brentwood Convalescent Home for Children, £15 13s. 4d.; Chelsea Hospital for Women Convalescent Home, St. Leonards, £58 15s.; Mrs. Gladstone's Convalescent Home, Mitcham, £111 12s. 6d.; Friendly Societies' Convalescent Home, Dover, £73 8s. 9d.; Hahnemann Convalescent Home, Bournemouth, £18 12s. 1d.; Hanwell Convalescent Home, £4 17s. 11d.; Hastings, Fairlight Sanatorium, £15 13s. 4d.; Herne Bay Baldwin-Brown Convalescent Home, £48 19s. 2d.; Homœopathic Hospital Convalescent Home, £2 18s. 9d.; Hemel Hempstead Convalescent Home, £36 4s. 7d.; Mary Yolland Home, £18 12s. 1d.; Mrs. Kitto's Convalescent Home, Reigate, £9 15s. 10d.; Police Seaside Home, Brighton, £60 14s. 2d.; St. Andrew's Convalescent Home, Clewer, £58 15s.; St. Andrew's Convalescent Home, Folkestone, £136 2s. 1d.; St. John's Home for Convalescent and Crippled Children, £24 9s. 7d.; St. Joseph's Convalescent Home, Bournemouth, £12 14s. 7d.; St. Leonards-on-Sea Convalescent Home for Poor Children, £127 5s. 10d.; Eversfield, St. Leonard's, £7 16s. 8d.; St. Mary's Convalescent Home, Birchington, £12 14s. 7d.; St. Mary's Convalescent Home, Broadstairs, £154 14s. 2d.; St. Mary's Convalescent Home, Shortlands, £16 12s. 11d.; St. Michael's Convalescent Home, Westgate, £17 12s. 6d.; Seaside Convalescent Hospital, Seaford, £48 19s. 2d.

TWENTY-SIX COTTAGE HOSPITALS.

Acton Cottage Hospital, £108 13s. 9d.; Beckenham Cottage Hospital, £94 19s. 7d.; Blackheath and Charlton Cottage Hospital, £112 12s. 1d.; Brentwood Cottage Hospital, £40 2s. 11d.; Bromley, Kent, Cottage Hospital, £115 10s. 10d.; Bushey Heath Cottage Hospital, £11 15s.; Canning Town Cottage Hospital, £134 2s. 11d.; Chislehurst, Sidcup and Cray Cottage Hospital, £90 1s. 8d.; Coldash Cottage Hospital, £47 19s. 7d.; East Ham Cottage Hospital, £52 17s. 6d.; Eltham Cottage Hospital, £67 11s. 3d.; Enfield Cottage Hospital, £91 1s. 3d.; Epsom and Ewell Cottage Hospital, £58 15s.; Hendon Cottage Hospital, £48 19s. 2d.; Hounslow Cottage Hospital, £62 13s. 4d.; Livingstone, Dartford Cottage Hospital, £128 5s. 5d.; Kingston, Victoria Hospital, £53 17s. 1d.; Molesey and Hampton Court Cottage Hospital, £14 13s. 9d.; Reigate and Redhill Hospital, £203 13s. 4d.; Sidcup Cottage Hospital, £34 5s. 5d.; Surbiton Cottage Hospital, £29 7s. 6d.; Teddington, £19 11s. 8d.; Tilbury (Passmore Edwards) Cottage Hospital, £48 19s. 2d.; Willesden Cottage Hospital, £112 12s. 1d.; Wimbledon (Nelson) Hospital, £49 18s. 9d.; Wood Green Cottage Hospital, £87 2s. 11d.

ELEVEN INSTITUTIONS.

Florence Nightingale Hospital for Invalid Gentlewomen, £136 2s. 1d.; St. Saviour's Hospital and Nursing Home, £87 2s. 11d.; Invalid Asylum, Stoke Newington, £47; Firs Home, Bournemouth, £14 13s. 9d.; St. Columba's Hospital, £293 15s.; St. Luke's House, Pembridge Square, £131 4s. 2d.; Santa Claus Home, Highgate, £76 7s. 6d.; Royal Mineral Water Hospital, Bath, £24 9s. 7d.; Winifred House, Holloway, £54 16s. 8d.; Highgate Convalescent Home, £44 1s. 3d.; Holt Church Sanatorium, £2 18s. 9d.

FORTY-ONE DISPENSARIES.

Battersea Provident Dispensary, £146 17s. 6d.; Billingsgate Dispensary, £47 19s. 7d.; Brixton, etc., Dispensary, £29 7s. 6d.; Buxton Street Dispensary, £9 15s. 10d.; Camberwell Provident Dispensary, £44 1s. 3d.; Childs Hill Provident Dispensary, £4 17s. 11d.; City Dispensary, £65 12s. 1d.; Clapham General and Provident Dispensary, £21 10s. 10d.; Eastern Dispensary, £31 6s. 8d.; East Dulwich Provident Dispensary, £45 0s. 10d.; Farringdon General Dispensary, £36 4s. 7d.; Finsbury Dispensary, £55 16s. 3d.; Forest Hill Provident Dispensary, £33 5s. 10d.; Greenwich Provident Dispensary, £24 9s. 7d.; Hampstead Provident Dispensary, £37 4s. 2d.; Islington Dispensary, £36 4s. 7d.; Islington Medical Mission, £47 19s. 7d.; Kilburn, Maida Vale, and St. John's Wood Dispensary, £33 5s. 10d.; Kilburn Provident Medical Institution, £44 1s. 3d.; London Dispensary, Spitalfields, £12 14s. 7d.; London Medical Mission, Endell Street, £83 4s. 7d.; Margaret Street, for Consumption, £42 2s. 1d.; Metropolitan Dispensary, £24 9s. 7d.; Mildmay Medical Mission Dispensary, £16 12s. 11d.; Notting Hill Provident Dispensary, £19 11s. 8d.; Paddington Provident Dispensary, £20 11s. 3d.; Public Dispensary, £42 2s. 1d.; Queen Adelaide's Dispensary, £10 15s. 5d.; Royal General Dispensary, £22 10s. 5d.; Royal Pimlico Provident Dispensary, £19 11s. 8d.; St. George's, Hanover Square, Dispensary, £22 10s. 5d.; St. John's Wood Provident Dispensary, £34 5s. 5d.; St. Marylebone General Dispensary, £73 8s. 9d.; St. Pancras and Northern Dispensary, £36 4s. 7d.; Stamford Hill, Stoke Newington Dispensary, £47; Wandsworth Common Provident Dispensary, £7 16s. 8d.; Westbourne Provident Dispensary, £11 15s.; Western Dispensary, £40 2s. 11d.; Western General Dispensary, £70 10s.; Westminster General Dispensary, £26 8s. 9d.; Woolwich Provident Dispensary, £29 7s. 6d.

THIRTY-THREE NURSING ASSOCIATIONS.

Belvedere, Abbey Wood, £7 9s.; Brixton, £44 14s.; Central St. Pancras, £29 16s.; Charlton and Blackheath, £7 9s.; Chelsea and Pimlico, £29 16s.; Hackney, £51 3s.; Hammersmith, £74 10s.; Hampstead, £22 7s.; Isleworth, £14 18s.; Kensington, £67 1s.; Kilburn, £7 9s.; Kingston, £37 5s.; Lambeth Road (Catholic), £7 9s.; Metropolitan (Bloomsbury), £51 3s.; St. Olave's (Bermondsey), £29 16s.; Paddington and Marylebone, £59 12s.; Plaistow, £59 12s.; Plaistow (Maternity), £89 8s.; Ponders End, Enfield, etc., £14 18s.; Rotherhithe, £14 18s.; Shoreditch, £59 12s.; Sick Room Helps Society, £29 16s.; Sidcup, £7 9s.; Silvertown, £14 18s.; South London (Battersea), £59 12s.; Southwark, £37 5s.; South Wimbledon, £7 9s.; Tottenham, £22 7s.; Westminster, £29 16s.; Woolwich, £51 3s.; East London, £119 4s.; North London, £74 10s.; Ranyard Nurses, £511 1s.

